# Community-forming traits play role in effective colonization of plant-growth-promoting bacteria and improved plant growth

**DOI:** 10.3389/fpls.2024.1332745

**Published:** 2024-03-12

**Authors:** Devashish Pathak, Archna Suman, Pushpendra Sharma, Krishnan Aswini, Venkadasamy Govindasamy, Shrikant Gond, Rana Anshika

**Affiliations:** Division of Microbiology, Indian Council of Agricultural Research (ICAR)-Indian Agricultural Research Institute, New Delhi, India

**Keywords:** biofilm, colonization, microbial community, microcosm, plant growth promoting traits, plant microbe interaction

## Abstract

Community-forming traits (CFts) play an important role in the effective colonization of plant-growth-promoting bacterial communities that influence host plants positively by modulating their adaptive functions. In this study, by considering plant-growth-promoting traits (PGPts) and community-forming traits (CFts), three communities were constructed, *viz.*, SM1 (PGPts), SM2 (CFts), and SM3 (PGPts+CFts). Each category isolates were picked up on the basis of their catabolic diversity of different carbon sources. Results revealed a distinctive pattern in the colonization of the communities possessed with CF traits. It was observed that the community with CFts colonized inside the plant in groups or in large aggregations, whereas the community with only PGPts colonized as separate individual and small colonies inside the plant root and leaf. The effect of SM3 in the microcosm experiment was more significant than the uninoculated control by 22.12%, 27.19%, and 9.11% improvement in germination percentage, chlorophyll content, and plant biomass, respectively. The significant difference shown by the microbial community SM3 clearly demonstrates the integrated effect of CFts and PGPts on effective colonization *vis-à-vis* positive influence on the host plant. Further detailed characterization of the interaction will take this technology ahead in sustainable agriculture.

## 1 Introduction

In the face of global challenges such as climate change, use of high concentration chemicals, and increasing food demand, sustainable agriculture practices have gained paramount importance. One such sustainable approach is the utilization of plant-growth-promoting bacteria (PGPB), which exhibit characteristics like nutrient solubilization, phytohormone production, and antagonism against pathogens (Javed et al., 2021; Sharf et al., 2021). Selecting the most effective isolates that can colonize effectively in the plant rhizosphere is the crucial step in designing the bioinoculants for integrated nutrient management approach (Oo et al., 2020) to enhance crop productivity while reducing the reliance on chemical fertilizers and pesticides (Cao et al., 2023).

PGPB is a diverse group of soil-dwelling and endophytic bacteria that inhabit the rhizosphere and endosphere of plants (Thakur et al., 2023), where they form intricate interactions with their host plants and contributes to their growth and overall well-being (Shoaib et al., 2020). Harnessing the full potential of PGPB involves not only identifying beneficial bacterial isolates but also their interactions within the complex microbial communities formed in the rhizosphere (Bulgarelli et al., 2013; Trivedi et al., 2020). The intricate communities are characterized by a myriad of interactions (Backer et al., 2018). Successful microbial communities are developed by the individual microbial partners possessing certain community-forming traits (CFts) such as biofilm formation, exopolysaccharide (EPS) production, and others like motility and PHB production. These traits contribute to the establishment of stable and cooperative environments that enhance the survival and functionality of individual microbes in various ecological niches (Wu et al., 2019; Karygianni et al., 2020; Hung et al., 2023). The role of CFts were least understood in plant growth and development.

Besides all these activities of microbial isolates, there is more to reveal about the impact of key dominant genera, which play a major role in community formation (Aswini et al., 2023; Yuan et al., 2023). The presence of key dominant genera in a community may mimic as synthetic natural community (Großkopf and Soyer, 2014; Shayanthan et al., 2022). The concept of synthetic communities (syncoms) has gained prominence as a tool for deciphering the synergistic effects of different microbial species on plant health (Liu et al., 2022; Hao et al., 2023).

Various studies conducted in the past discussed about the plant growth promoters and their effect over various plant species (Bhat et al., 2023; Carreiras et al., 2023). There is a gap to see the integrated effect of the CFts over PGP bacterial activity and their cumulative effect on plant growth and development as well as the practical construction and evaluation of synthetic communities (SMs). This study investigates how these CFts contribute to the establishment of beneficial interactions within the rhizosphere and their subsequent impact on wheat plant growth. We hypothesized that the cumulative effect of CFts and PGP bacterial community works in a better way than only PGP bacterial community.

By assembling PGP bacteria with known CFts, this study aimed to elucidate how these synthetic communities influence wheat plant growth in a controlled microcosm environment by shedding light on these complex interactions. We aimed to contribute valuable insights into sustainable agricultural practices that can mitigate the challenges of an ever-changing world and pave the way for increased crop yields and food security.

## 2 Materials and methods

### 2.1 Bacterial isolates

Two sources for bacterial isolation were taken: one was from the culture bank of our lab, and another was done by isolation process from wheat rhizospheric soil and endosphere. A total of 187 cultures of wheat and maize was taken from the culture bank of our lab (Marag and Suman, 2018; SaiPrasad et al., 2021), Division of Microbiology, ICAR–IARI, New Delhi, India. Working stock and slants were made in nutrient broth and nutrient agar and kept at 4°C for further work. Root and rhizospheric soil of the wheat variety HD2967 were taken from the field of the Division of Genetics, ICAR–IARI, New Delhi (28° 36′ 50″ N:77° 12′ 32″ E), Nashik (20° 04′ 59″ N:74° 07′ 00″ E), Samastipur (25° 51′ 39″ N:85° 46′ 56″ E), Indore (22° 43′ 31″ N:75° 51′ 55″ E), and Shimla (31° 06′ 12″ N:77° 10′ 20″ E), and HD2833 from Coimbatore (11° 22′ 12″ N:06° 48′ 00″ E) and were collected on the given coordinates mentioned. Samples were taken aseptically to the laboratory and stored at 4°C until further analysis.

### 2.2 Isolation of bacteria from wheat root endosphere and rhizosphere

Bacteria were isolated from wheat endosphere and rhizosphere of varieties HD2967 and HD2833. Isolation was done by using nutrient agar and tryptic soy agar. First, rhizospheric soil was separated carefully from root samples using sterile brush under aseptic conditions. One gram of soil was serially diluted in 10 ml of saline water (0.8%, w/v), and fourth to fifth dilutions were used to isolate the rhizospheric bacteria. For endospheric bacterial isolation, root samples were surface sterilized with 1% NaOCl for 1 min followed by 30 s of ethanol treatment followed by three times washing with sterile water (Aswini et al., 2023). One gram of root sample was macerated in sterile mortar and pestle, and similar dilutions were used for isolation of endophytic bacteria. The plates were incubated at 28 ± 2°C for 3 days and observed regularly. The isolates were selected based on their morphology, which were purified (Abd El Gawad et al., 2010; Taye et al., 2021) and preserved in 30% glycerol at −20°C till further study.

### 2.3 Screening for community-forming traits

CFts are the traits which require bacterial quorum sensing mechanism to show their optimality, e.g., biofilm formation and EPS production. Some other traits are also there, which are essential in a community to survive, *viz*., motility and PHB production. In a community, a proper division of carbon source or nutrients is also essential, which requires different metabolic diversity pattern among isolates (Wei et al., 2020; Dal Bello et al., 2021).

#### 2.3.1 Biofilm formation

All bacterial isolates were checked for the ability to form biofilm on a microtiter plate (O'Toole, 2011). Biofilm production was done with the crystal violet staining method, where bacterial isolates were first grown separately to reach OD = 1, then reinoculated into the ELISA 96-well plate with biofilm assay medium, i.e., M63 minimal medium [KH_2_PO_4_ (22 mM), K_2_HPO_4_ (40 mM), and (NH_4_)_2_SO_4_ (15 mM)] supplemented with MgSO_4_ (1 mM), casamino acid (0.5%), and glucose (0.2%). Freshly prepared bacterial culture and medium were mixed in 1:100 (V/V) ratios in three replicates. Optical density (OD) of grown bacterial culture was taken with respect to the OD of the biofilm or adhered cell to the surface of the wall for 24, 48, and 72 h of growth. Biofilm OD was taken by staining the adhered cell with crystal violet followed by solubilization of the stained cell with 30% acetic acid solution.

#### 2.3.2 Exopolysaccharides production

EPS production was quantified with Congo red binding assay (Quintieri et al., 2020). Prior to the activity, equal cell density was maintained of each isolate and grown to the OD = 1 in Luria–Bertani (LB) broth. Each isolate was reinoculated in M63 broth for 72 h. After the end of the incubation time, the broth was centrifuged at 10,397 rpm for 5 min, and the pellets of the culture were again suspended in 1 ml of 40 µg ml^−1^ of Congo red solution. Suspended cultures were incubated for 90 min with vigorous shaking for 10 min at each interval of 15 min for three times. The samples were again centrifuged at 9,600 rpm for 5 min, and OD of the supernatant was taken at 490 nm. The final OD revealed the amount of EPS produced, which was the OD of the blank subtracted from the OD of the inoculated. The amount of EPS production was obtained by matching the OD with the standard curve of different concentrations of Congo red.

#### 2.3.3 Motility

Motility of the isolates was performed in the nutrient semi-solid agar media supplemented with 1% TTC. The freshly grown cultures were stabbed straight into the test tube within the medium and were incubated at 35 ± 2°C for 24 h (Kelly and Fulton, 1953). As cells start to replicate and move, they turned TTC into the red colored compound triphenylformazan, and the extent of motility was observed with the expanded colored area inside the tube.

#### 2.3.4 Polyhydroxy butyrate

Polyhydroxy butyrate (PHB) is an important compound produced by bacterial isolates, which was used to observe in an abundance of sugar content with minimum competition. PHB is stored within the bacterial cell for use in stressful conditions. The PHB producers were identified with the help of Sudan black dye (60 mg of dye in 200 ml of 70% ethanol). The freshly grown cultures in 1% glucose supplemented nutrient broth for 48 h at 35± 2°C were spotted over the nutrient agar plate (also supplemented with 1% glucose) incubated at 35 ± 2°C for 24 h to allow proper growth. Sudan black dye was poured over the culture plate to cover it all. After 1 min, the extra stain was drained out of the plate and was washed off with the help of 96% ethanol (Williamson and Wilkinson, 1958).

#### 2.3.5 Metabolic diversity

HiCarbo™ Kit (KB 009, HiCarbohydrate™ kit) and HiAssorted™ Biochemical Test Kit (KB002) of HiMedia was used to determine the utilization of various carbon sources as carbohydrates and carboxyl type, along with this some amino acids and enzymatic activities (*viz.*, esculin hydrolyses, urease, β-galactosidase activity, nitrate reductase, and phenylalanine deaminase) by isolates. The kit consists of three strips A, B, and C with all biochemical compounds. A freshly prepared (with 0.5 OD at 620 nm) 50-µl culture of all bacterial isolates was inoculated over the substrate bound inside each well of the strips and incubated at 35± 2°C for 24 h. The change in color showed the positive test for the particular kind of substrate (Kumar et al., 2021).

### 2.4 Screening for plant growth promoting traits

#### 2.4.1 Nutrient mobilization

A set of 294 selected wheat and maize rhizospheric and endospheric microbials was screened for the fixation of nitrogen and solubilization of phosphorus, potassium, zinc, and calcium carbonate. Nitrogen fixation was done with the stab culture in semisolid JNF (Jensen’s nitrogen-free) medium followed by ARA estimation by gas chromatography [GC-2014, Shimadzu, Japan with a flame ionization detector (FID)]. Phosphorus, potassium, and zinc solubilization was observed over the plate of Pikovskaya media (with 0.5% tricalcium phosphate), Aleksandrov media (containing 0.2% of potassium aluminosilicate), and nutrient agar fortified with 0.1% insoluble zinc compound (ZnO), respectively, as described in Verma et al. (2015). Calcite solubilization was observed over the agar plate containing (g 100 ml^−1^) CaCO_3_ (0.5), dextrose (1.0), [NH_4_]_2_SO_4_ (0.05), KCl (0.02), MgSO_4_.7H_2_O (0.1), yeast extract (0.5), and agar (1.5) with pH 7 (Rana et al., 2015). The plates with respective isolates showing solubilization zones for macro- and micro-nutrients were considered as positive for respective traits. For the quantification of phosphorus, Pikovskaya media (50 ml) were inoculated with each freshly grown positive isolate and incubated at 28 ± 2°C at 180 rpm; the supernatant centrifuged at 8,000 rpm was collected to estimate phosphorus colorimetrically by molybdenum-blue assay (Liu et al., 2014).

#### 2.4.2 Phytohormones and ACC deaminase

All purified isolates were screened for various phytohormones, *viz.*, gibberellic acid (GA_3_) and indole acetic acid (IAA). GA_3_ estimation was done as described by Brown and Burlingham (1968). Isolates were inoculated in 20 ml of LB broth and kept overnight for incubation at 30 ± 2°C. After incubation for 7 days, the culture broth was centrifuged at 8,000 rpm for 10 min; 15 ml of supernatant was mixed vigorously with 2 ml of zinc acetate solution followed by mixing of 2 ml of potassium ferrocyanide followed by centrifugation at 8,000 rpm for 10 min and mixing of 5 ml HCl (30%); and the final solution was incubated for 45 min. After the period of incubation, the OD was observed at 254 nm. Five milliliters of 5% HCl was used as blank. The concentration of GA in each culture medium was determined by comparison with the standard GA curve. Indole 3-acetic acids (IAA) estimation was done by the growing of culture in nutrient broth fortified with 1% tryptophan and incubation for 15 days at 28 ± 2°C. The uninoculated tube was maintained as control. The culture broth was centrifuged at 8,000 rpm for 10 min, 2 ml of the supernatant obtained was mixed with 4 ml of Salkowski reagent (50 ml, 35% perchloric acid; 1 ml 0.5 FeCl_3_) and two drops of concentrated orthophosphoric acid; the mixture was incubated in dark condition for 30 min for color development. The developed pink color depicts IAA production qualitatively; for quantitative estimation, OD was taken at 530 nm by using a spectrophotometer (Halo DB-20S). The concentration of IAA was estimated with the help of a standard curve of 10–100 μg ml^−1^ following Bric et al. (1991).

ACC-deaminase activity and reduction in ACC was described by Glick (1995) and Li et al. (2011). Accordingly, freshly grown isolates were centrifuged in nutrient broth at 8,000 rpm for 10 min, and the obtained cell pellet was washed with DF medium as described in Glick (1995). The washed pellet was again suspended in DF-ACC medium with a final ACC concentration of 3.0 mmol l^−1^ for 24 h incubation. The incubated culture was again centrifuged with 0.1 ml supernatant and diluted 10 times with DF medium. The final volume was mixed with double volume of ninhydrine reagent (50 mg ninhydrin + 1.5 mg ascorbic acid + 30 ml ethylene glycol, equal volume of 1 mol l^−1^ citrate buffer was mixed before use (pH 6.0). The DF medium was considered as blank. Deep purple color to light purple color reveals the extent of ACC deaminase activity, which was measured at 570 nm. The result was compared with the standard curve of ACC in DF medium for quantified value.

#### 2.4.3 Biocontrol traits

The biocontrol activity of microbes involves the traits to suppress or control the growth of harmful or pathogenic microbes through the production of siderophores, hydrogen cyanide (HCN), and ammonia (NH_3_). The selected isolates were screened for these traits and antagonism test against pathogenic fungi. Siderophore qualitative estimation was done with the change in the color of CAS agar plate from blue to yellow. The quantification of the siderophore was done for qualitatively selected isolates from the plate assay. The selected isolates were inoculated in LB broth. When the cells attained a cfu count of 10^8^ (approximate), the broth was centrifuged, and pellets were discarded. Then, 0.5 ml of the supernatant was mixed with 0.5 ml of CAS reagent prepared as mentioned by Schwyn and Neilands (1987). After 20 min, observations were taken in the form of OD at 630 nm with the help of a spectrophotometer. The reading was quantified in terms of percentage siderophore unit (psu) by Shelley (1994). Ammonia production was identified with the help of Nessler’s reagent, which was added (1 ml) in the well-grown culture broth (10 ml); change in the color from yellow to brown or brickish red confirmed the production of NH_3_ (Cappuccino and Sherman, 1992). HCN quantitative estimation was done by colorimetric analysis of the changed picric-acid-soaked filter paper. In this regard, the change in the color of alkaline picric-acid-soaked filter paper was eluted in double-distilled water (ddH_2_O), and OD was taken at 625 nm (Rijavec and Lapanje, 2016). Different concentrations of potassium ferrocyanide compound were used to prepare standard curve for actual cyanide content, which was used to calibrate the HCN produced by bacterial isolates.

Plate assay was carried out for antagonism against pathogenic fungi. All selected isolates were tested for the antagonistic ability against various pathogens., e.g., *Tilletia*, *Bipolaris*, *Puccinia*, and *Fusarium* over the PDA plates to observe the positive and negative interaction with the pathogenic fungi by bacterial isolates compared with the control plate of the respective fungus (Sharma et al., 2023).

### 2.5 Identification of potential isolates

All isolates were selected based on their ability to show better CFts and PGPts. The isolation of genomic DNA was done using the Quick-DNA™ Fungal/Bacterial DNA Miniprep Kit and subjected to a gel run to check the quality of purified DNA. A partial sequence of the 16S rRNA gene was amplified with forward pA (F)—(5′AGAGTTTGATCCTGGCTCAG-3′) and reverse pH (R)—(5′AAGGAGGTGATCCAGCCGCA-3′) as the universal primer using a PCR thermocycler (HiMedia Prima-96). PCR products were delivered to HiMedia for Sanger sequencing and purification. The obtained raw forward and reverse sequences were further processed for contig formation using BioEdit. Misaligned reads in between the contigs, which were used for identification, was checked with the help of Decipher 3.0. To identify, NCBI-BLAST of the final contigs were done and matched with the data of microorganisms having the highest percentage of similarity (>97%) in the NCBI database.

### 2.6 Designing microbial communities

#### 2.6.1 Compatibility test

The test of compatibility among isolates was checked over the plate by cross-streaking method. All isolates were cross-streaked one by one so that every isolate could cross another at once with everyone. The isolates that grown together at junction were considered compatible and were taken for further grouping (Pugsley and Oudega, 1987). Along with this, growth patterns of all individual isolates were correlated with the mixed culture (called interaction analysis, not shown here), which revealed about the interaction between the individual isolates.

#### 2.6.2 Development of microbial communities

Each isolate with positive compatibility was grown separately in a 50-ml flask containing 10 ml nutrient broth. Each isolate was allowed to grow up to 1 OD. The selection of microbes for each community was based on a bonitur scale for CFts, PGPts, and CFts+PGPts. The isolates with maximum bonitur value for plant-growth-promoting traits were grouped and termed “PGPts” or SM1. Similarly, the isolates having high bonitur value for community-forming traits were grouped and termed “CFts” or SM2. In addition, the compatible isolates having both the traits (PGPts and CFts) were grouped and termed “CFts+PGPts” or SM3. The final volume (100 ml) of each community was prepared by mixing 10 ml of each isolate, where per milliliter of culture broth contains approximately 1×10^8^ cells.

### 2.7 Root colonization study

Surface-sterilized wheat seeds with 1% NaOCl for 60 s followed by ethanol (70%) for 30 s followed by three times wash with sterile double-distilled water (ddH_2_O) were used to check the colonization potential of the isolates. Each group of the community, which was previously grown separately to reach 1 OD, was allowed to inoculate the prepared seeds for 1.5 h. Inoculated or treated seeds were pressed gently over the soft agar plate (0.8% agar) (five seeds/plate) inside the laminar air flow, to stick over the plates, and were covered with the upper lid. The triplicates of uninoculated (control) and inoculated plates were incubated for 5−7 days in an upright condition at 20°C till the seedling stage. Prior to the visualization, the plates with proper seedlings were flooded with TTC stain, containing TTC (1.5 g l^−1^) and malic acid (625 mg l^−1^) in potassium phosphate (0.05 M) buffer (pH 7.0) (Thomas and Sekhar, 2014) for 10 h. Visualization of the leaf and root tissue section was done under the bright field microscope (De winter) after proper washing of the sample with sterile ddH_2_O.

### 2.8 Microcosm study on plant growth

To evaluate the effect of microbial communities, a pot experiment was taken in the glass house of the Division of Microbiology, ICAR-IARI, New Delhi. The weather condition was 15°C–20°C with 6 h of bright sunny day. The pot (20 cm diameter and 20 cm height) used was presanitized with 1% NaOCl followed by washing with sterile distilled water. The pot was filled with 3 kg of sterile (3 days of sterilization at 120°C 103.42 kPa for 1 h) rooting medium (soil: vermiculite: sand, 3:1:1 ratio). The required fertilizer dose (RDF for wheat 120:60:60 NPK kg ha^−1^) was mixed with the soil according to the weight of soil in treatments before sowing. Split nitrogen dose application was maintained before and after the germination of wheat seedlings. Overnight grown culture with similar OD was mixed in equal volume according to their communities (CFts, PGPts, and PGPts+CFts). Sterile seeds were treated with each community separately for 2–3 h followed by drying in laminar air flow. The pot experiment was set up in a completely randomized design (CRD) in triplicate having the following treatments: T1–100% RDF, T2–50% RDF + PGP, T3–50% RDF + CFts, T4–50% RDF + PGP + CFts, and T5–50% RDF as negative control. Under this experiment plant biometric data like plant height (cm), root length (cm), and chlorophyll content (nmol cm ^-1^) were taken at 60 DAS and 90 DAS. Along with this, germination percentage and plant dry weight were taken at initiation and harvesting stage, respectively.

### 2.9 Statistics

Analysis of plant biometric data was done over the OPSTAT for ANOVA followed by Duncan’s *post hoc* analysis of microcosm experiments, whereas for the visualization of the metabolic pattern of different carbon sources utilization, construction of constellation tree was done for each isolate by hierarchical cluster method in JMP software (v17.0).

## 3 Results

### 3.1 Isolation of bacteria

Among six wheat samples from different regions of the country, the range of bacterial load in rhizospheric soil was 5.4×10^5^ to 8.7×10^5^ cfu g^−1^ in which rhizospheric the soil sample from Shimla had the highest count followed by that from Delhi. The range of root endophytic bacterial count was between 2.4×10^5^ and 5.1×10^5^ cfu g^−1^, in which Shimla had highest count followed by Coimbatore (**Table 1**). A total of 107 morphotypes were selected in which 53 were rhizospheric soil isolates and 54 were root endophytic isolates (**Supplementary Table S1**).

**Table 1:** Microbial count and selected morphotypes from wheat samples.

Site	Coordinates	Microbial count CFU g^−1^		Selected bacterial morphotypes	
		Rhizosphere	Root endosphere	Rhizosphere	Root endosphere
Nashik	20° 04′ 59″ N:74° 07′ 00″ E	6.6×10^5^	3.5 × 10^5^	13	11
Delhi	28° 36′ 50″ N:77° 12′ 32″ E	8.3×10^5^	3.3×10^5^	6	8
Indore	22° 43′ 31″ N:75° 51′ 55″ E	5.4×10^5^	2.4×10^5^	9	7
Coimbatore	11° 22′ 12″ N:06° 48′ 00″ E	6.8×10^5^	4.8×10^5^	11	6
Shimla	31° 06′ 12″ N:77° 10′ 20″ E	8.7×10^5^	5.1×10^5^	4	14
Samastipur	25° 51′ 39″ N:85° 46′ 56″ E	7.7×10^5^	4.0×10^5^	10	8

### 3.2 Community-forming traits of isolates

**Figure 1:**
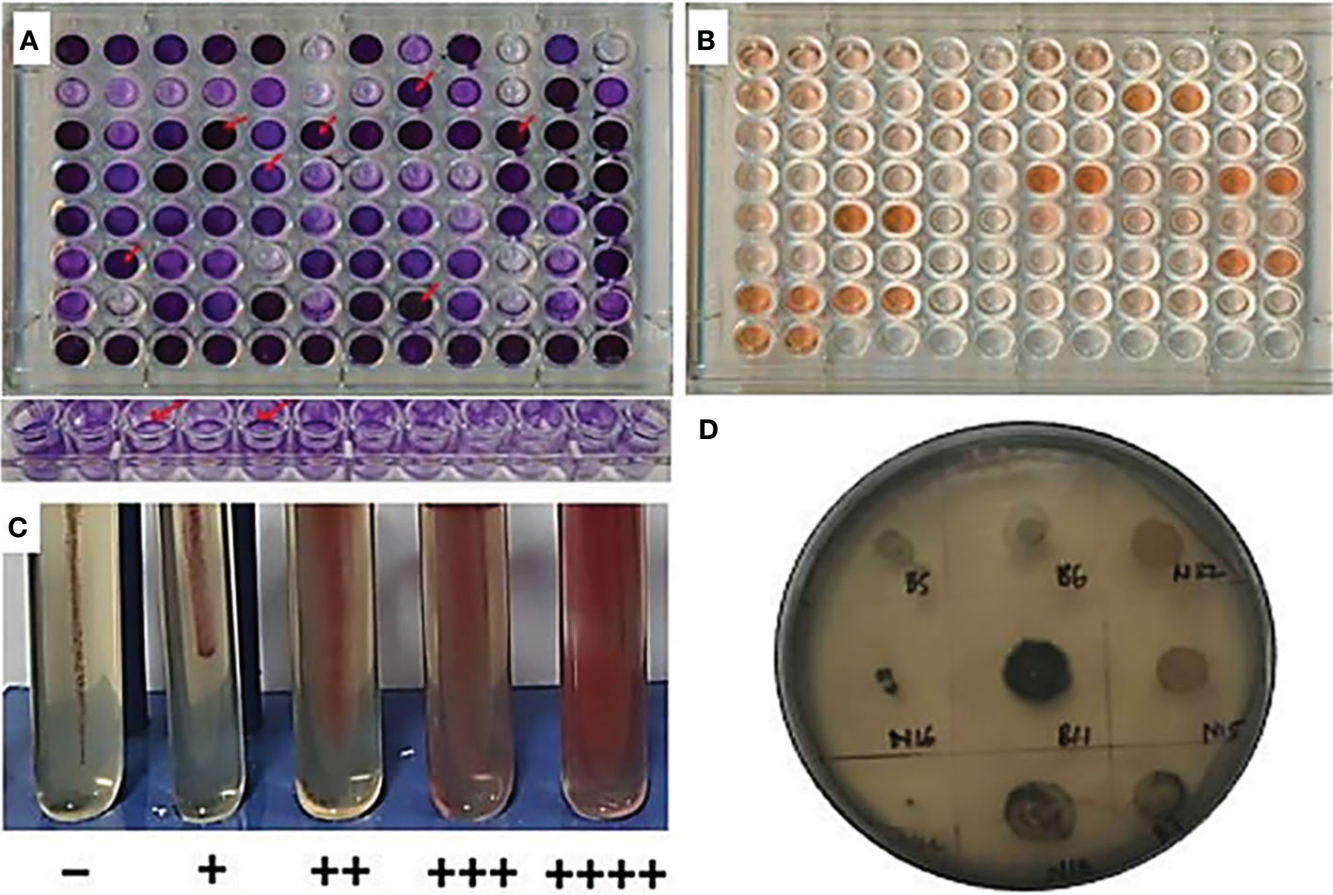
Community forming traits. **(A)** Crystal violet-stained biofilm in microtiter plates; **(B)** Congo red dye method for EPS; **(C)** motility potential of bacterial isolates in nutrient agar with 1% TTC; and **(D)** Sudan Black dye stained PHB-positive isolate.

General CFts, which include EPS, biofilm production, PHB production, and motility, were considered in this experiment (**Figure 1**). The range of EPS production is from 2.281 ± 0.021 µg ml^−1^ to 28.72 ± 0.630 µg ml^−1^ with mean average production of 14.88 µg ml^−1^. Among all isolates, the highest producer of the EPS is DWE-1 followed by NWP-12. Whereas in biofilm production, the range of the biofilm formation was found from 0.003 OD to 3.88 OD, among 294 isolates, seven showed the highest production of biofilm, which was >3.8 OD. Among all isolates, there were five levels of motility shown, 0%, 25%, 75%, and 100% based on the coverage area of the test tube, in that 29 isolates were showing maximum motility, which covered 100% of the area of the tube in 24 h. In PHB production, a total of 11 isolates retained the stain of Sudan black dye in their colony and had not wash off from 95% ethanol. Among all 294 isolates, the top 17 cultures were screened based on their bonitur scale and were further kept for community purposes (**Table 2**; **Supplementary Table S2**).

**Table 2:** Community-forming traits of selected bacterial isolates.

Isolates	EPS(µg ml^−1^)	Biofilm	Motility^*^	PHB	Bonitur^#^
**D-1**	28.720 ± 0.630	3.867 ± 0.009	25	+	10
**NWP-12**	18.747 ± 0.331	2.820 ± 0.057	100	−	9
**PHM5-29**	10.810 ± 0.021	3.803 ± 0.019	100	−	9
**BWR-11**	12.213 ± 0.259	0.797 ± 0.003	100	**+++**	**9**
**PZ-28**	20.071 ± 0.252	1.877 ± 0.011	75	−	**8**
**PHM5-27**	14.520 ± 0.312	3.683 ± 0.038	75	−	8
**PHM5-36**	14.520 ± 0.339	3.483 ± 0.101	25	−	8
**NWE-2**	25.420 ± 0.396	2.080 ± 0.006	25	−	8
**NWE-11**	23.720 ± 0.333	3.467 ± 0.009	25	−	8
**NWR-14**	16.873 ± 0.202	3.587 ± 0.050	25	−	8
**PHM5-33**	24.027 ± 0.324	3.877 ± 0.030	**−**	+	8
**PC4-44**	18.557 ± 0.232	1.893 ± 0.033	75	−	8
**SHZ-30**	18.230 ± 0.171	1.789 ± 0.013	100	−	8
**PHM5-34**	21.351 ± 0.101	2.781 ± 0.017	25	+	8
**DWE-3**	26.317 ± 0.383	0.997 ± 0.003	50	−	7
**IWE-1**	19.483 ± 0.051	1.550 ± 0.021	75	−	7
**NWR-13**	18.663 ± 0.378	3.060 ± 0.021	**−**	**+**	**7**
**PHM5-9**	11.587 ± 0.283	0.970 ± 0.01	50	+++	7
**SHZ-37**	20.787 ± 0.398	2.890 ± 0.047	25	−	7

Each value indicates the mean of three replicates with ± SD; + and – indicate the presence or absence of particular trait.

*Area covered in a test tube by motile isolate, i.e., 25%, 50%, 75%, and 100%.

^#^Bonitur scale range:

Exopolysaccharides (EPS): <5 = 0; 5–10 = 1; 10–15 = 2; 15–20 = 3; 20–25 = 4.

Biofilm: 0–1 = 0; 1–2 = 1; 2–3 = 2; 3–4 = 3.

Motility: 25% = 1; 50% = 2; 75% = 3; 100% = 4.

Polyhydroxy butyrate (PHB): each + sign marked as 1 point.

### 3.3 Plant-growth-promoting traits of isolates

Among all 294 isolates, K-solubilization was seen in 58 isolates, and the range of index was from 0.75 to 3.04, in which NWR-15 was showing the highest solubilization index followed by NWR-14. P-solubilization ability ranged between 4.2 ppm and 44.61 ppm, where NWPZ-60 showed the highest solubilization of phosphorus, i.e., 44.61 ppm followed by PHM5–21 (37.45 ppm). Zinc solubilization was seen in 32 isolates with range of 0.678–2.764 of PC4–29. Coming to the Ca complex, a total of 63 isolates out of 294 were showing solubilization, in which 13 isolates were having maximum solubilization potential of Ca complex (**Table 3**).

**Table 3:** Plant-growth-promoting traits (PGPts) of selected bacterial isolates.

Isolates	Nutrient solubilization/fixation					Phytohormone production/inhibition of ACC			Biocontrol activity			Antagonistic activity against pathogenic fungi			Bonitur^*^
	N (nmoles of C_2_H_4_ mg protein^−1^ h^−1^)	PS (ppm)	KS (SI − K)	CaCO_3_ solubilization	ZnS (SI − Zn)	GA (ppm)	IAA (ppm)	ACC deaminase activity (mmol of ACC conversion)	Sid (psu)	Ammonia production	HCN production (ppm)	*Bipolaris*	*Puccinia*	*Alternaria*	
**IWE-6**	4.90 ± 0.05	44.61 ± 0.83	1.90 ± 0.01	++++	2.36 ± 0.13	4.73 ± 0.04	17.83 ± 0.37	1.65 ± 0.01	9.00 ± 0.05	++++	2.313 ± 0.02	−	++	−	38
**NWE-9**	6.05 ± 0.01	20.53 ± 0.35	1.62 ± 0.01	++++	−	11.06 ± 0.16	20.63 ± 0.04	2.18 ± 0.04	10.49 ± 0.21	++++	26.18 ± 0.42	+	++	−	34
**NWPZ-60**	5.15 ± 0.16	44.61 + 0.34		++++	−	12.40 ± 0.10	4.43 ± 0.11	2.03 ± 0.00	61.72 ± 0.52		2.38 ± 0.01	−	+	−	30
**PHM5-22**	8.17 ± 0.12	18.21 ± 0.01	1.18 ± 0.01	+++	−	4.06 ± 0.02	13.23 ± 0.11	2.40 ± 0.02	12.31 ± 0.18	++++	31.05 ± 0.18	−	++	+	29
**PHM5-35**	−	29.61 ± 0.15	0.182 ± 0.01	+++	−	5.40 ± 0.11	13.33 ± 0.34	2.68 ± 0.06	11.50 ± 0.21	+++	35.65 ± 0.22	−	−	−	29
**PHM5-9**	−	20.29 ± 0.38		++	−	4.40 ± 0.01	−	1.92 ± 0.04	10.60 ± 0.20	++++	63.78 ± 0.48	−	+	+	28
**PC4-59**	−	−	2.15 ± 0.13	+++	1.68 ± 0.00	5.06 ± 0.00	37.33 ± 0.17	2.20 ± 0.00	11.50 ± 0.27	+++	36.78 ± 0.42	−	−	−	27
**NWR-15**	1.87 ± 0.00	22.85 ± 0.00	3.04 ± 0.38	+	−	8.06 ± 0.19	45.53 ± 0.38	2.38 ± 0.01	23.99 ± 0.51	++	2.05 + 0.01	−	+++	−	24
**PHM5-73**	−	20.61 ± 0.21	1.67 ± 0.22	+++	−	5.06 ± 0.10	12.43 ± 0.23	2.38 ± 0.03	13.40 ± 0.14	++++	32.85 ± 0.25	−	−	−	26
**PHM5-74**	−	20.93 ± 0.19	1.70 ± 0.04	+++	−	8.39 ± 0.17	19.53 ± 0.04	2.26 ± 0.05	11.10 ± 0.06	++++	27.78 ± 0.17	−	−	−	25
**IWE-7**	4.75 ± 0.12	15.61 ± 0.08	−	++	2.16 ± 0.31	4.73 ± 0.03	18.43 ± 0.15	1.64 ± 0.00	9.90 ± 0.01	++++	1.91 ± 0.01	+	++	−	23
**SWE-12**	5.53 ± 0.03	22.53 ± 0.02	−	++	−	6.73 ± 0.01	22.13 ± 0.05	2.14 ± 0.03	14.71 ± 0.13	++	37.58 ± 0.30	−	−	−	23
**PHM5-59**	1.03 ± 0.01	15.65 ± 0.07	−	++	−	3.73 ± 0.01	15.83 ± 0.13	1.94 ± 0.03	−	+++	38.45 ± 0.34	−	−	−	19
**DWE-2**	9.16 ± 0.20	11.77 ± 0.07	2.39 ± 0.03	+++	−	6.06 ± 0.04	6.73 ± 0.01	1.41 ± 0.01	9.60 ± 0.12	++	2.85 ± 0.02	++	++	−	21
**BWR-15**	5.52 ± 0.13	26.05 ± 0.23	−	++	−	6.73 ± 0.19	22.83 ± 0.14	2.82 ± 0.02	14.00 ± 0.12	+	34.98 ± 0.28	−	−	−	21
**BWE-6**	2.11 ± 0.10	18.37 ± 0.12	−	+++	2.64 ± 0.01	4.06 ± 0.01	7.43 ± 0.02	2.24 ± 0.05	−	+++	9.98 ± 0.02	−	−	−	17
**NEP-56**	1.92 ± 0.00	10.97 ± 0.03	−	+++	−	7.06 ± 0.02	4.63 ± 0.03	2.03 ± 0.05	26.96 ± 0.32	++	15.51 ± 0.13	++	++	−	17

Each value indicates the mean of three replicates with ± SD, + and – indicates the presence or absence of characters.

*Bonitur scale range:

Nitrogen (N): <1 = 0; 1–2 = 1; 2–4 = 2; 4–6 = 3; 6–8 = 4; 8–9 = 5.

Phosphorus (P): <5 = 0; 5–10 = 1; 10–15 = 2; 15–20 = 3; 20–25 = 4; 25–30 = 5; 30–35 = 6; 35–40 = 7; 40–45 = 8.

Potassium (K): <0.6 = 0; 0.6 -1.2 = 1; 1.2–1.7 = 2; 1.7–2.2 = 3; 2.2–2.7 = 4; >2.7 = 5.

Siderophore (Sid): <1 = 0; 1–10 = 1; 10–20 = 2; 20–30 = 3; 30–40 = 4; 40–50 = 5; 50–60 = 6; 60–70 = 7.

Zinc solubilisation (Zn): <0.5 = 0; 0.5–1.0 = 1; 1.0–1.5 = 2; 1.5–2.0 = 3; 2.0–2.5 = 4.

Gibberellic acid (GA): <3 = 0; 3–6 = 1; 6–9 = 2; 9–12 = 3; 12–15 = 4; 15–18 = 5.

Indole acetic acid (IAA): <5 = 0; 5–10 = 1; 10–15 = 2; 15–20 = 3; 20–25 = 4; 25–30 = 5.

ACC deaminase activity: <0.5 = 0; 0.5–1 = 1; 1–1.5 = 2; 1.5–2 = 3; 2–2.5 = 4; 2.5–3 = 5.

HCN (Hydrogen Cyanide): 0–10 = 0; 10–20 = 1; 20–30 = 2; 30–40 = 3; 40–50 = 4; 50–60 = 5; 60–70 = 6; >70 = 7.

Ammonia/CaCO_3_/antagonistic activity: Each + sign mark 1 point.

SI − K/Zn, solubilization index of K/Zn [solubilization index = (colony diameter + diameter of halo zone)/colony diameter]; PS, phosphorus solubilization; KS, potassium solubilization; ZnS, zinc solubilisation.

**Figure 2:**
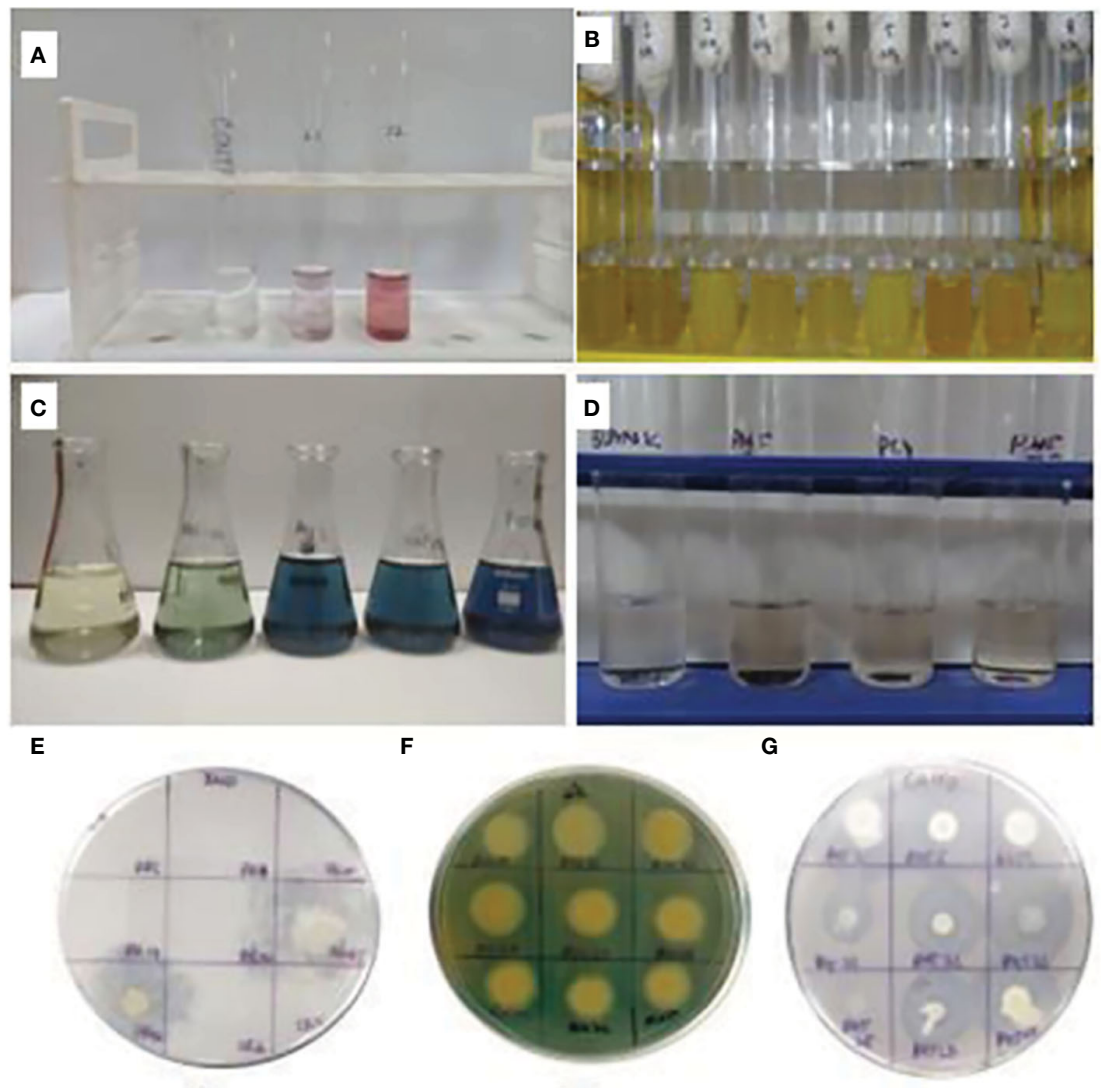
Plant-growth-promoting traits. **(A)** IAA production; **(B)** ammonia production; **(C)** phosphorus estimation; **(D)** GA activity; **(E)** Zn solubilization; **(F)** siderophore production; **(G)** CaCO_3_ solubilization.

In phytohormone production, the evaluation of isolates was done for GA and IAA. The production of GA and IAA was observed by the change in the color of the centrifuged culture broth with respective reagents (**Figure 2**). In the quantitative estimation of GA production test, out of 294 bacterial strains, 73 bacterial strains showed positive result in the range of 2.40–17.73 ppm. Among all 73 positive isolates, N14 was considered as the highest producer of GA. IAA production was shown by 77 isolates with minimum to maximum range of 0.5–53.73 ppm. Among all, PHM5-68 showed maximum production of IAA, i.e., 53.73 ppm, whereas ACC deaminase activity was observed from 0.05 ppm to 1.58 ppm, in which DWE-2 showed the maximum production among all (**Table 3**; **Supplementary Table S2**).

There are various traits for biocontrol mechanisms that control the growth of harmful or pathogenic microbes. In this study, HCN production, siderophore production, and ammonia production were taken as three important ways responsible for the biocontrol activity. HCN quantification was done to estimate the produced HCN from bacterial isolates. Among all 294 isolates, 87 isolates showed positive behavior for HCN production with a range of 0.58–63.2 ppm. The most potential isolate for HCN production among all isolates was PHM5-9 followed by TWR-9. Ammonia production was seen for 54 isolates from 294 isolates, in which 11 isolates showed higher production (++++) than others. In siderophore quantification, the range of siderophore production was from 8.04 ppm to 61.72 ppm produced from 68 isolates, where PZ-27 showed the highest production of siderophore at 72.06 ppm followed by PC4-29 (61.72 ppm) (**Table 3**).

Another biocontrol activity was assessed, i.e., antagonism against pathogenic fungi. The antagonistic activity of the isolates was observed against *Bipolaris sorokiniana*, *Fusarium graminearum*, *Puccinia striiformis*, and *Tilletia indica.* The bacterial isolates showing antagonistic activity did not allow any kind of pathogenic fungi to grow and degenerated the fungal mycelium. Among all 294 bacterial isolates, 58 isolates showed positive activity against the growth of pathogenic fungi. Among the 58 isolated, 16 (PC4-29, NH-3, NH-4, 5, NWP-9, NWP-11, NWP-12, NWP-14, NEP-22, PZ-23, PZ-24, SHZ-36, CZ-44, CZ-45, CZ-46, and DWE-1) showed better inhibition irrespective of the pathogenic fungi (**Table 3**; **Supplementary Figure S3**).

### 3.4 Selection of potential microbes

Among all 294 isolates, 187 isolates of culture bank and 107 were isolated. All isolates were first identified with CFts and then for PGPts. Out of 294 isolates, 136 isolates showed positive behavior of CFts, and another 67 isolates were positive for PGPts. Out of the two groups when matched together, 131 isolates showed both characteristics. To choose the top 10 isolates for all three groups, bonitur scaling was applied, which gave the ranking of each isolate according to their efficient multi-variate characteristics. The final three groups of isolates were identified, e.g., CFts (**Table 2**), PGPts (**Table 3**), and CFts+PGPts (**Table 4**), based on their peculiar multi-variate traits.

**Table 4:** Selected isolates showing both PGP and CFts.

Isolates	Community-forming traits (CFts)			Nutrient solubilization/fixation				Phytohormone production/inhibition of ACC			Biocontrol activity		Antagonistic activity against pathogenic fungi			
	EPS (µg ml^−1^)	Biofilm	Motility (%)	N (nmoles of C_2_H_4_/mg protein/h)	PS (ppm)	KS (SI − K)	ZnS (SI − Zn)	GA (ppm)	IAA (ppm)	ACC deaminase activity (mmol of ACC conversion)	Sid (psu)	HCN (ppm)	*Bipolaris*	*Puccinia*	*Alternaria*	*Tilletia*
**PC4-29**	17.64 ± 0.35	2.14 ± 0.04	75	7.79 ± 0.11	–	–	2.76 ± 0.16	5.07 ± 0.045	18.13 ± 0.04	2.20 ± 0.05	61.72 ± 0.76	37.91 ± 0.56	+	+	+	+
**PHM5-35**	24.84 ± 0.34	3.03 ± 0.01	25	–	29.61 ± 0.15	0.18 ± 0.012	–	5.40 ± 0.11	13.33 ± 0.34	2.68 ± 0.06	11.50 ± 0.21	35.64 ± 0.22	−	−	−	−
**NH-3**	18.18 ± 0.42	1.65 ± 0.01	75	–	8.49 ± 0.11	–	–	12.73 ± 0.31	14.83 ± 0.36	2.21 ± 0.00	54.31 ± 1.21	3.24 ± 0.03	+	+	+	+
**NWP-9**	19.11 ± 0.03	1.53 ± 0.03	75	7.09 ± 0.21	15.81 ± 0.15	–	–	3.73 ± 0.05	23.83 ± 0.58	2.26 ± 0.05	56.33 ± 0.64	40.11 ± 0.91	+	+	+	+
**NWPZ-60**	12.67 ± 0.07	1.17 ± 0.02	50	5.15 ± 0.04	44.61 ± 0.83	–	–	12.40 ± 0.15	4.43 ± 0.06	2.03 ± 0.00	61.72 ± 0.38	2.38 ± 0.04	−	−	−	−
**IWE-6**			50	4.90 ± 0.05	27.41 ± 0.64	1.90 ± 0.01	2.37 ± 0.14	4.73 ± 0.04	17.83 ± 0.37	1.65 ± 0.01	9.00 ± 0.05	2.31 ± 0.02	−	++	−	−
**NH-1**	19.87 ± 0.11	3.88 ± 0.01	25	7.40 ± 0.18	7.97 ± 0.20	–	–	7.73 ± 0.18	23.53 ± 0.08	2.39 ± 0.02	52.28 ± 1.17	40.24 ± 0.20	+	++	−	−
**PC4-31**	16.42 ± 0.39	2.34 ± 0.05	75	5.29 ± 0.00		1.65 ± 0.27	2.53 ± 0.54	4.73 ± 0.07	14.43 ± 0.27	1.92 ± 0.02	20.60 ± 0.37	39.11 ± 0.02	−	−	−	−
**SHZ-30**	18.23 ± 0.22	1.79 ± 0.04	100	4.06 ± 0.05	10.09 ± 0.25	–	–	5.40 ± 0.00	13.73 ± 0.28	2.07 ± 0.03	53.26 ± 0.36	1.04 ± 0.01	+	++	−	−
**NWR-14**	16.87 ± 0.39	3.88 ± 0.01	50	5.33 ± 0.02	17.97 ± 0.43	2.65 ± 0.40	–	17.73 ± 0.03	0.50 ± 0.01	2.26 ± 0.02	13.20 ± 0.08	0.98 ± 0.00	+	++	−	−
**PHM5-34**	21.35 ± 0.17	2.78 ± 0.02	25	3.26 ± 0.01	27.25 ± 0.14	–	–	3.73 ± 0.03	14.03 ± 0.28	2.51 ± 0.01		35.04 ± 0.56	−	−	−	−
**PHM5-21**	13.69 ± 0.3	1.30 ± 0.00	25	–	37.45 ± 0.39	–	–	4.40 ± 0.01	13.63 ± 0.24	2.17 ± 0.05	14.00 ± 0.26	63.11 ± 1.08	−	+	+	−
**NH-2**	13.46 ± 0.14	1.04 ± 0.01	75	–	8.93 ± 0.04	–	–	2.40 ± 0.03	16.83 ± 0.31	2.69 ± 0.01	36.78 ± 0.24	43.18 ± 0.11	+	+	−	−
**PC4-43**	18.67 ± 0.15	1.69 ± 0.04	75	–	–	2.38 ± 0.41	1.68 ± 0.27	4.73 ± 0.12	14.53 ± 0.34	2.22 ± 0.03	27.00 ± 0.247	30.38 ± 0.21	−	−	−	−
**PZ-27**	14.47 ± 0.31	1.83 ± 0.02	25	–	9.57 ± 0.09	–	–	6.73 ± 0.07	17.03 ± 0.17	2.44 ± 0.00	72.06 ± 0.29	1.24 ± 0.02	−	−	−	−
**BWE-4**	18.23 ± 0.05	0.32 ± 0.00	50	4.44 ± 0.10	36.85 ± 0.31	1.73 ± 0.31	–	5.73 ± 0.12	23.43 ± 0.18	2.54 ± 0.04	9.00 ± 0.17	0.84 ± 0.01	−	−	−	−
**PZ-28**	20.07 ± 0.13	1.87 ± 0.00	75	3.33 ± 0.04	11.81 ± 0.08	–	–	9.06 ± 0.20	16.23 ± 0.38	2.44 ± 0.00	34.16 ± 0.71	1.11 ± 0.01	−	−	−	−
**NWR-13**	18.66 ± 0.37	3.06 ± 0.02	–	5.73 ± 0.07	11.29 ± 0.41	–	–	3.73 ± 0.01	18.13 ± 0.24	1.79 ± 0.02	11.22 ± 0.10	17.91 ± 0.02	−	+++	−	−
**NWE-9**	11.88 ± 0.27	–	50	6.05 ± 0.08	20.53 ± 0.37	0.16 ± 0.01	–	11.07 ± 0.22	20.63 ± 0.04	2.18 ± 0.04	10.50 ± 0.15	26.18 ± 0.42	+	++	−	−
**IWE-7**	–	–	75	4.75 ± 0.12	15.61 ± 0.09	–	2.15 ± 0.31	4.73 ± 0.03	18.43 ± 0.15	1.64 ± 0.00	9.90 ± 0.02	1.91 ± 0.01	+	++	−	−
**DWE-1**	28.72 ± 0.63	3.87 ± 0.00	25	–	–	–	–	–	–	2.71 ± 0.05	–	39.11 ± 0.51	+	+	+	+
**PC4-59**	9.31 ± 0.02	1.27 ± 0.04	–	4.41 ± 0.18	15.56 ± 0.38	2.00 ± 0.05	0.67 ± 0.05	4.40 ± 0.04	17.93 ± 0.05	1.84 ± 0.03	15.19 ± 0.36	36.78 ± 0.42	−	+	−	−

Each value indicates the mean of three replicates with ± SD, + and – indicates the presence or absence of characters.

SI − K/Zn, Solubilization index of K/Zn [solubilization index = (colony diameter + diameter of halo zone)/colony diameter]; PS, phosphorus solubilization; KS, potassium solubilization; ZnS, zinc solubilisation.

### 3.5 Metabolic diversity pattern

**Figure 3:**
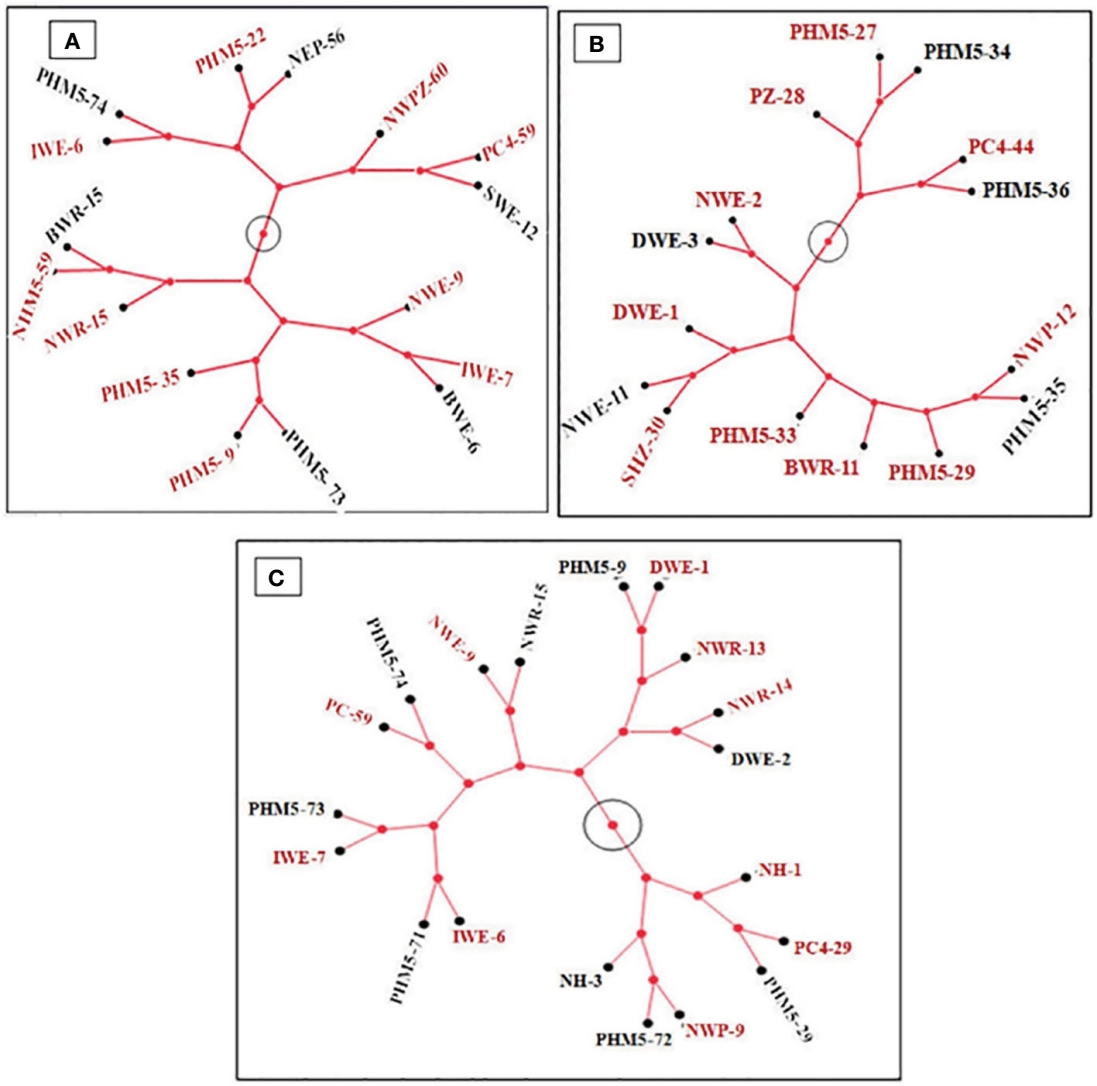
Constellation tree pattern of metabolic diversity of selected isolates for community formation: **(A)** SM1 (PGPt); **(B)** SM2 (CFt); **(C)** SM3 (PGPt+CFt). Isolate numbers in red font in a clad are selected for community development.

The selected isolates were ensured for their diverse metabolic pattern for the utilization of different carbon sources. The grouping or community was made considering the diverse carbon utilization. In the cladogram of PGP community, results of top 16 bacterial isolates were selected based on their carbon utilization pattern (**Figure 3A**). The bacterial isolates that form the same clade, e.g., PHM5-22 and NEP-56, PC4-59 and SWE-12, IWE-7 and BWE-6, PHM5-73 and PHM5-9, BWR-15 and PHM5-59, and IWE-6 and PHM5-74, shared similar behavior in the carbon utilization pattern. These bacterial isolates may hinder each other’s growth or may compete for the similar food source. Among top CFt isolates, five clades (PHM5-35 and NWP-12, NWE-11 and SHZ-30, NWE-2 and DWE-3, PHM5-36 and PC4-44, and PHM5-27 and PHM5-34) were formed (**Figure 3B**). In the formation of PGPts+CFts group, the isolates were again checked for further carbon utilization to ensure the least competition while forming any community, in that eight clades (PHM5-9 and DWE-1, NWR-14 and DWE-2, PC4-29 and PHM5-29, NWP-9 and PHM5-72, IWE-7 and PHM5-73, IWE-6 and PHM5-71, PC4-59 and PHM5-74, and NWE-9 and NWR-15) were formed, which shared the similar carbon utilization pattern (**Figure 3C**).

### 3.6 Compatibility and construction of community

The bacterial isolates selected for PGPt communities were streaked across to check the compatibility over the plate. All the bacterial isolates were compatible with each other and were selected for community formation, considering the fact to take one isolate based on the higher ranking of bonitur scale from each clade of metabolic diversity tree, to minimize the competition. Each community was made up of equal number of isolates, i.e., 10. In PGPts, community consists of IWE-6, NWE-9, IWE-7, PC4-59, NWR-15, NWPZ-60, PHM5-9, PHM5-35, PHM5-22, and PHM5-59 isolates, named as SM1. CFts were constructed with DWE-1, NWP-12, BWR-11, PZ-28, SHZ-30, PHM5-27, NWE-2, PHM5-33, PHM5-29, and PC4-44, which was named as SM2, whereas PGPts+CFts was constituted with PC4-29, NWP-9, NWR-14, NH-1, NWR-13, IWE-6, new-9, IWE-7, DWE-1, and PC4-59 named as SM3. The selection of one isolate from each clade was based upon the superiority in the bonitur scale among the two. The final three communities constructed, e.g., SM1 (PGPts), SM2 (CFts), and SM3 (CFts+PGPts), were based on their peculiar multi-variate traits ready for treatments (**Table 5**).

**Table 5:** Phylogeny of selected bacterial isolates for community development.

Selected isolates	Accession no. of isolate	Nearest type strain	Accession no. of type strain	Percentage similarity
PC4-59	OR739532	*Klebsiella variicola* strain F2R9	NR_025635	99.79
PHM5-22	OR739535	*Klebsiella variicola* strain JCM1662	NR_025635	99.64
NWE-9	OR739519	*Enterobacter cloacae* subsp. *dissolvens* strain LMG 2623	NR_044978	99.79
PHM5-35	OR739539	*Acinetobacter pittii* DSM 21653 strain ATCC 19004	NR_117621	99.93
NWR-15	OR739523	*Pseudomonas laurentiana* strain GSL-010	NR_179898	99.86
IWE-6	OR739517	*Stenotrophomonas pavanii* strain ICB89	NR_116793	98.80
PHM5-9	OR739534	*Bacillus haynesii* strain NRRL B-41327	NR_157609	98.24
IWE-7	OR739524	*Bacillus stercoris* JCM30051	NR_180796	99.65
NWPZ-60	MT184855	*Bacillus subtilis* DSM10	AJ276351	99.75
PHM5-59	OR739541	*Acinetobacter calcoaceticus* strain NCCB22016	NR_042387	99.93
NWE-2	OR739518	*Bacillus stercoris* JCM30051	NR_180796	99.72
PHM5-29	OR739537	*Kosakonia cowanii* JCM 10956	NR_025566	99.10
SHZ-30	MT184842	*Bacillus velezensis* strain FZB42	NR_075005	99.71
DWE-1	OR739513	*Bacillus amyloliquefaciens* NBRC15535	NR_180225	99.44
PZ-28	MT184840	*Bacillus velezensis* strain CBMB 205	NR_116240	99.73
PHM5-33	KT149755	*Bacillus amyloliquefaciens* MPA 1034	NR_117946	99.79
PHM5-27	OR739536	*Bacillus altitudinis* strain 41KF2b	NR_042337	99.72
NWP-12	MT184824	*Bacillus aerius* strain 24K	NR_118439	99.93
BWR-11	OR739510	*Bacillus proteolyticus* strain MCCC 1A00365	NR_152692	99.68
PC4-44	OR739530	*Brevibacillus reuszeri* strain NBRC 15719	NR_113802	99.9
NWR-14	OR739522	*Bacillus amyloliquefaciens* strain MPA1034	NR_180225	99.93
PC4-29	OR739527	*Bacillus amyloliquefaciens* BCRC 11601	NR_180197	99.58
NH-1	MT672532	*Bacillus altitudinis* strain 41KF2b	NR_042337	99.72
NWP-9	MT184821	*Pantoea agglomerans* strain NCTC 9381	NR_114735	98.78
NWR-13	OR739521	*Stenotrophomonas pavanii* strain LMG25348	NR_118008	99.46

### 3.7 Identification

In all three communities, each of the 10 selected isolates (except the already identified), PCR products were outsourced for Sangers sequencing of the V3–V4 region to HiMedia for identification. The results of the matched contigs were compared on NCBI database with more than 97% of similarity. All the selected isolates of the SM1 community were identified as *Enterobacter cloacae* subsp. *dissolvens* strain LMG 2623, *Stenotrophomonas pavanii* strain ICB89, *Bacillus stercoris* JCM30051, *Klebsiella varicola* strain F2R9, *Pseudomonas laurentiana* strain GSL-010, *Bacillus subtilis* DSM10, *Bacillus haynesii* strain NRRL B-41327, *Acinetobacter pittii* DSM 21653 strain ATCC 19004*, Klebsiella variicola* strain JCM1662, and *Acinetobacter calcoaceticus* strain NCCB22016, respectively, for NWE-9, IWE-6, IWE-7, PC4-59, NWR-15, NWPZ-60, PHM5-9, PHM5-35, PHM5-22, and PHM5-59 isolates with more 97% similarity (**Table 5**; **Supplementary Figure S2**). The isolates in the SM2 community were identified as *B. amyloliquefaciens* NBRC15535, *B. aerius* strain 24K, *B. proteolyticus* strain MCCC 1A00365, *B. velezensis* strain CBMB 205, *B. velezensis* strain FZB42, *B. altitudinis* strain 41KF2b, *B. stercoris* JCM30051, *B. amyloliquefaciens* MPA 1034, *Kosakonia cowanii* JCM 10956, and *Brevibacillus reuszeri* strain NBRC 15719, respectively, for DWE-1, NWP-12, BWR-11, PZ-28, SHZ-30, PHM5-27, NWE-2, PHM5-33, PHM5-29, and PC4-44 (**Table 5**; **Supplementary Figure S2**). The isolates for SM3 community were identified as *B. amyloliquefaciens* BCRC 11601, *Pantoea agglomerans* strain NCTC 9381, *B. amyloliquefaciens* strain MPA1034, *B. altitudinis* strain 41KF2b, *S. pavanii* strain LMG25348, *S. pavanii* strain ICB89, *Enterobacter cloacae* subsp*. dissolvens* strain LMG 2623, *B. stercoris* JCM30051, *B. amyloliquefaciens* NBRC15535, and *Klebsiella variicola* strain F2R9, respectively, for PC4-29, NWP-9, NWR-14, NH-1, NWR-13, IWE-6, NWE-9, IWE-7, DWE-1, and PC4-59 (**Supplementary Figure S2**; **Table 5**).

### 3.8 Root colonization of the communities

The trans-section view of root and leaf samples was observed under the bright field microscope. In root samples, the microbial group at 18 h of inoculation was colonized more over the root tip and inside the secondary root hairs. It was like they were entering into the root endosphere for all three communities, but the bacterial colonies were absent for the leaf. At 48 h of inoculation, the root cortex region was fully colonized with the microbial communities, and some colonization was also observed inside the leaf. At 72 h of incubation, the colonization was observed more for the root and leaf endosphere compared with the colonization at 48 h. Microbial colonization was observed inside the cell and in intercellular spaces. There were some differences in colonization behavior observed between the three communities. The colonization of the SM2 and SM3 communities was higher, and they were in large aggregates compared with the scattered single cells of the SM1 community (**Supplementary Figure S1**).

### 3.9 Plant biometric assay

**Figure 4:**
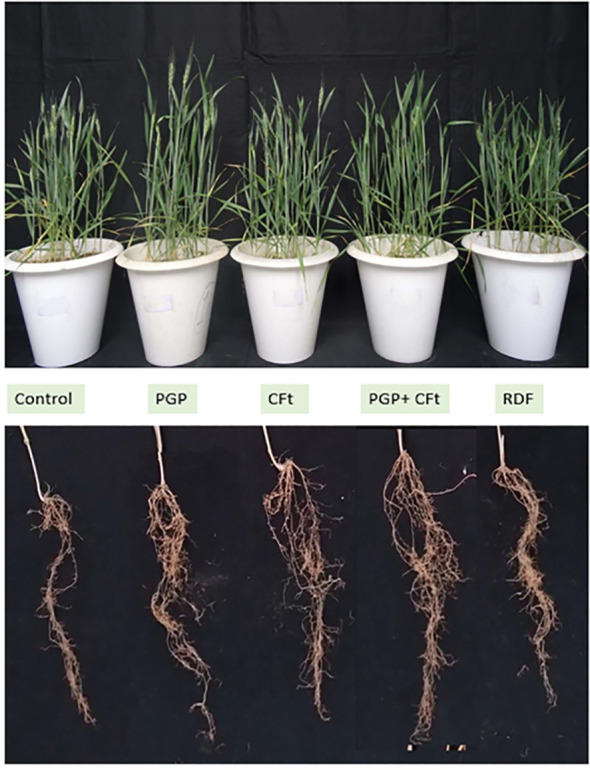
Effect of developed bacterial communities’ inoculation on wheat plant growth.

All prepared communities were evaluated by the pot experiments with wheat plants (**Figure 4**). Plant biometric assays like germination percentage, root length (60 DAS and 90 DAS), shoot length (60 DAS and 90 DAS), plant dry weight (at 90 DAS), and chlorophyll content (60 and 90 DAS) were evaluated under the glass house or semi-control condition. All communities SM1, SM2, and SM3 were compared with the negative control and 100% RDF-treated pots. In germination percentage, T5 (100% RDF) and T4 (SM3 + 50% RDF) showed the highest germination percentage and insignificantly differed with each other. The treatment receiving SM3 + 50% RDF (T4) showed 28% higher germination compared with the control (T1) (**Table 6**). The chlorophyll content of the plant at 60 DAS and 90 DAS was evaluated with the help of SPAD. The chlorophyll content was significantly higher in T4 than in other treatments T1, T2, and T3 by 37.35%, 21.5%, and 25.14%, respectively, at 60 DAS (**Table 6**). Plant dry matter was calculated at 90 DAS, which when compared to the negative control with biological treatments, dry weight was 9.11% higher in T4 followed by T2 and T3 by 4.8% and 3.12%, respectively (**Table 6**). Among the microbial treatments, all were significantly different from each other with higher plant biomass in T4, which was 4.3% and 6% higher than in T2 and T3, respectively. Among all treatments, the T4 (SM3) result was better than the rest of the microbial treatments and negative control irrespective of the plant traits, which showed that there is some cumulative effect of CFts over PGPts.

**Table 6:** Effect of microbial communities on plant growth and development in microcosm experiment.

Treatment		Germ%	CHL (SPAD reading)		RL (cm)		SL (cm)		PB (mg)
			60 DAS	90 DAS	60 DAS	90 DAS	60 DAS	90 DAS	
T1	Control	70.20 ± 0.53^c^	30.60 ± 0.66^c^	31.58 ± 0.53^d^	12.90 ± 0.13^e^	17.53 ± 0.44^d^	24.43 ± 0.20^e^	30.27 ± 0.46^e^	351.77 ± 1.25^d^
T2	SM1(PGP)	83.80 ± 2.14^b^	34.59 ± 0.36^b^	35.63 ± 0.04^bc^	15.32 ± 0.27^d^	22.99 ± 0.36^c^	33.06 ± 0.84^c^	35.40 ± 0.35^c^	368.78 ± 2.31^bc^
T3	SM2 (CFt)	85.75 ± 0.45^b^	33.61 ± 0.40^b^	34.61 ± 0.07^c^	16.78 ± 0.36^c^	22.11 ± 0.05^c^	30.11 ± 0.25^d^	32.69 ± 0.31^d^	362.15 ± 4.71^c^
T4	SM3 (PGPt+CFt)	90.15 ± 1.36^a^	42.03 ± 0.66^a^	40.52 ± 0.18^a^	19.32 ± 0.37^b^	25.56 ± 0.56^b^	37.45 ± 0.66^b^	40.94 ± 0.36^b^	383.91 ± 8.79^b^
T5	RDF	91.38 ± 0.04^a^	35.64 ± 0.89^b^	36.24 ± 0.57^b^	21.32 ± 0.33^a^	27.47 ± 0.23^a^	40.14 ± 0.29^a^	43.30 ± 0.61^a^	417.46 ± 10.43^a^
C.D.		3.75	1.99	1.15	0.98	1.18	1.65	1.36	20.93
SE(m)		1.17	0.62	0.36	0.31	0.37	0.51	0.43	6.56
SE(d)		1.66	0.88	0.51	0.43	0.52	0.73	0.61	9.26
C.V.		2.4	3.06	1.74	3.09	2.78	2.70	2.04	3.01

Each value in a column represents mean of three replications with ± SD. Same superscript alphabets do not differ significantly at *p* = 0.05 by Duncan’s multiple range test.

Germ%, germination percentage; RL, root length; SL, shoot length; PB, plant biomass; DAS, days of showing.

## 4 Discussion

Wheat, being one of the most pivotal and staple crops, is cultivated across India, covering approximately 29.8 million hectares. Over the past decade, there has been a notable surge in wheat production, with the country’s output escalating from 94.57 million metric tons in the 2012–2013 period to 112.18 million tons in 2022–2023 (Srivastava et al., 2023). Its demand is rising in countries undergoing urbanization and industrialization. In addition to serving as a significant source of starch and energy, wheat also provides substantial quantities of various essential elements crucial for health, including protein, essential vitamins (particularly B vitamins), dietary fiber, and beneficial phytochemicals (Shewry and Hey, 2015). Consequently, it draws the attention of researchers from various domains to enhance both the productivity and quality of grains. Utilizing traditional biofertilizers is a viable approach for the sustainable growth of wheat. However, it comes with certain drawbacks. For instance, employing a single-strain fertilizer may have limited effectiveness in diverse environments due to factors such as competition and predation in exotic surroundings and challenges in achieving proper colonization (Somasegaran and Bohlool, 1990; Bradáčová et al., 2019). These issues can be addressed by employing a consortium, but a significant challenge lies in ensuring compatibility among the members of the consortium and their persistence in different environments. Therefore, concerning microbiology, it is important to advance research in developing wheat endophytic and rhizospheric bacterial communities, which is certain in the colonization of wheat plant and mimics the natural community that also contributes to crop growth and development.

This experiment utilized endophytes and rhizospheric isolates from wheat and maize to develop bacterial communities. Community construction was based on CFts and their PGPts. It was observed that, in addition to the PGPts, various CFts were also present in bacterial isolates, which are essential for community integrity and functioning (Wong et al., 2021). The significant CFts include biofilm synthesis, exopolysaccharide production, polyhydroxybutyrate production, and, to some extent, motility. Motility is a factor that enables beneficial microbes to be attracted towards root exudates and joins the community based on their compatibility, indirectly contributing to community formation. Motility also facilitates the occupation of nutrient-rich niches in the vicinity of the plant, enriching the space with beneficial microbes (Palma et al., 2022). In CFts, several mentioned traits were observed in which the range of EPS was found from 11 to 28.72 µg ml^−1^, which satisfied with the findings of Mancuso Nichols et al. (2004) and Chambi et al. (2021). Comparing the OD of biofilm formation, the findings of Simoes et al. (2007) and Thomen et al. (2017) were comparable to our observations where biofilm ranges between 0.1 and 4 OD at 540 nm. Motility was measured in the percentage coverage of the test tube by motile cells in a stab culture; a similar experiment was done by Murinda et al. (2002), which matched with our observation. PHB-producing bacteria preserve the extra carbohydrate present in the niche as intracellular reservoir to tackle with the adverse condition. This study covered qualitative analysis of PHB production with Sudan black dye. Black-stained colonies were considered as positive isolates for PHB production, which was matched with the findings of Robert and Iyer (2018).

Combining isolates with potential CFts and PGPts represents a novel approach towards developing plant probiotics. Various scientists have conducted extensive research on the plant-growth-promoting traits of endophytes and rhizospheric bacteria, such as nutrient solubilization, phytohormone production, and antifungal activity (Eid et al., 2021; Sharma et al., 2023). In our study, the plant growth parameters were examined both qualitatively and quantitatively, where nitrogen and phosphorus results came in the range of 4–9.16 nmol C_2_H_4_ mg protein^−1^ h^−1^ and 6.78–44.61 ppm, respectively, which aligned with the findings of other workers (Gull et al., 2004; Widdig et al., 2019) in the previous studies. The range of results for phytohormone, *viz*., IAA and GA_3_ analysis, was 4.43–45.53 ppm and 3.73–12.40 ppm, which was related with the findings of Karadeniz et al. (2006) and Restu et al. (2019), respectively. Siderophore analysis was done with the use of CAS reagent and supernatant of culture broth. The range of total siderophore production was 9.0–61.72 psu. Previous analysis of siderophore production was carried by various scientists (Murakami et al., 2021; Newman, 2023), which satisfied our results.

Including the general screening traits (e.g., PGPts and CFts), microbial communities were developed in this experiment to mimic naturally occurring communities. Previously, in the laboratory, strategies for creating synthetic microbial communities based on functional and/or abundance approaches have been devised (Suman et al., 2022). To validate this, a wet lab synthesis of microbial communities was conducted using key species from heat-sensitive varieties of wheat. These communities were developed and validated in both controlled and field experiments (Aswini et al., 2023). While in this study, the key genera were identified from the selected isolates, their dominance was also confirmed with the dominant genera identified from the metagenome (BioProject-PRJNA944920, PRJNA 954583, and PRJNA 1065154) submitted by the lab in NCBI database. From these projects, the abundant genera found concerning wheat endophytes were *Bacillus*, *Stenotrophomonas*, *Pseudomonas*, *Acinetobacter*, *Enterobacter*, *Clostridium*, and *Xanthomonas*. The previous experiment, carried out by Tkalec et al. (2022), supported our findings, where they observed abundance of *Bacillus* species in their samples. The bacterial genera *Clostridium* and *Xanthomonas* were excluded from community formation due to their pathogenic nature for plants and animals. The isolates of key dominant species were integrated into the communities based on their multifunctional traits, placing them in their respective community categories (SM1, SM2, and SM3).

After grouping all isolates according to their functional traits, they were exposed to different carbon sources and other compounds to assess their resource requirements. Previous studies by various other scientists (Uroz et al., 2007; Tzamali et al., 2011; Kumar et al., 2021) have shown that in a community, there is a proper distribution of food sources (Passari et al., 2016). In this study, isolates in a clade (which showed same carbon source utilizing pattern) possessing higher bonitur scale for PGPts and CFts were selected for the development of communities. Furthermore, they were checked for compatibility through cross-inoculation on agar plates, which aligned with the findings of Murillo-Roos et al. (2022). All 46 screened bacterial isolates were identified using Sanger sequencing of the V3–V4 region of 16S rDNA. The results indicated that *Bacillus* was the predominant genus, followed by *Enterobactor*, and others including *Pantoea*, *Microbacterium*, *Stenotrophomonas*, and *Pseudomonas*. This result is consistent with the findings of Suman et al. (2020); Alaylar et al. (2021) and Sharma et al. (2023) where most of the rhizospheric and endophytic culturable microbes were dominated by the Bacilli group.

The formed communities were inoculated onto wheat seedlings grown on water agar plates to observe the colonization behavior of communities. Bacterial colonization was assessed using TTC staining, and in our experiments, colonization ability was evaluated at three time intervals: 24 h, 48 h, and 72 h. Initially, colonization was observed at the tip of the secondary root, followed by colonization inside the root and leaf. The observations regarding the colonization of bacterial communities in root and leaf at different periods were consistent with the findings of various workers (Remus et al., 2000; Timmusk et al., 2005; Thomas and Sekhar, 2014). The new behavior of the bacterial community was observed, e.g., formation of aggregation in the community possessed community-forming traits and more single isolated bacterial colony was found in the community, which had only PGPts. The aggregation of the community inside the plant system defines the strength of integrity among individual isolates; being together inside the plant system may impact better than the single isolated bacterial strain. There are various such findings that observed that a bacterial community and consortium showed better results than the application of a single isolated strain (Bradáčová et al., 2019; Santoyo et al., 2021).

In the microcosm experiment, the evaluation of formed communities (PGPt, CFt, and PGPt+CFt) was conducted in sterile soil to assess various plant growth attributes. In our experiments, the germination percentage was found to increase with the application of PGPt+CFt community and did not significantly differ from the treatment with a full dose of fertilizer application. This finding aligns with similar research conducted by Pacheco et al. (2021) and Hussain et al. (2021). The observation of root length was highest in the T1 treatment, followed by T4, at both stages of plant growth. The root growth in T4 was higher compared to the other treatments and the control. Similar results were observed in studies by Khan et al. (2022) and Sukeerthi et al. (2020). Regarding shoot length, the pot with a full dose of fertilizer application showed the highest growth compared to the other treatments, followed by the T4 treatment. In both stages of plant growth, the T4 treatment exhibited better growth than the other two microbial treatments, showing a 54% and 34% increase over the control, which was consistent with the findings of Marag and Suman (2018). In terms of chlorophyll content, treatment T4 consistently showed the highest levels, regardless of the date of observation and treatments. However, the chlorophyll content did not significantly increase at later stages of plant growth compared to the other traits. This finding is consistent with an experiment conducted by Reynolds et al. (2000), where they observed a plateau in the increment of chlorophyll content in wheat plants at the terminal and anthesis times. This supports our result, indicating that at a certain stage, the chlorophyll content of the plant does not increase further. Additionally, a higher chlorophyll content was found in the microbial treatment, which is also consistent with the findings of Khalilzadeh et al. (2018). Plant dry matter was also influenced by the application of microbial treatments, with T4 being only 8% lower than the full dose of fertilizer application and 9.1% higher than the control. This result was aligned with the findings of Bilal et al. (2017) and de Matos Nascimento et al. (2020) in their experiments. A higher plant biomass and other growth attributes were found in the treatments, where the community was showing both the traits of PGP and CF; these findings bound us to consider the effect of community-forming traits, which increases the efficacy of plant-growth-promoting traits of various isolates.

## 5 Conclusion

Community-forming traits play a crucial role in facilitating effective rhizosphere colonization and recruitment by plants as endophytes. The integration of efficient PGP bacteria with additional isolates possessing community-forming traits has shown great potential as a probiotic inoculant for enhancing chlorophyll content and plant biomass and further the yield increment. Further exploration of the interactive mechanisms in field trials with the selected communities will open up new strategic avenues for developing innovative bio-inoculants to promote sustainable agriculture.

## Data availability statement

The original contributions presented in the study are included in the article/**Supplementary Material**, further inquiries can be directed to the corresponding author/s.

## Author contributions

DP: Data curation, Formal analysis, Investigation, Methodology, Validation, Visualization, Writing – original draft. AS: Conceptualization, Data curation, Funding acquisition, Investigation, Resources, Supervision, Validation, Writing – original draft, Writing – review & editing. VG: Methodology, Funding acquisition, Resources, Validation, Writing – review & editing. PS: Methodology, Formal analysis, Software, Visualization, Writing – original draft. KA: Investigation, Methodology, Resources, Software, Writing – review & editing. SG: Funding acquisition, Investigation, Methodology, Resources, Supervision, Writing – original draft. RA: Investigation, Methodology, Writing – original draft.
