# Supplementary material for: Community-forming traits play role in effective colonization of plant-growth-promoting bacteria and improved plant growth
Source: Front Plant Sci. 2024 Mar 12;15:1332745. doi: 10.3389/fpls.2024.1332745 (PMC10963436; doi:10.3389/fpls.2024.1332745)
Supplement: Supplementary file 1 [file DataSheet_1.docx]

**Community Forming traits play role in effective colonization of Plant Growth Promoting bacteria and improved plant growth**

Devashish Pathak, Archna Suman*, Pushpendra Sharma, Aswini K, V Govindasamy, Shrikant Gond and Anshika

*Corresponding Author

Division of Microbiology, ICAR-Indian Agricultural Research Institute, New Delhi, India-110012


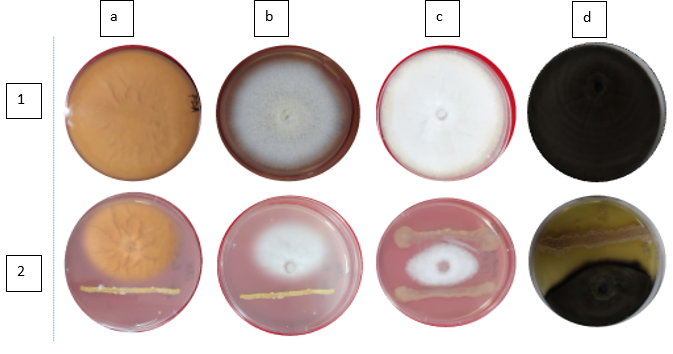


**Fig. S1. Effect of antagonistic bacterial isolates on harmful fungus. 1) and 2) show the control plate and treated plate respectively. a) inhibition of the *Puccina* growth, b) inhibition of *Alterneria* growth, c) inhibition of *Tilitia* growth, and d) inhibition of *Bipolaris* growth compared with the control.**


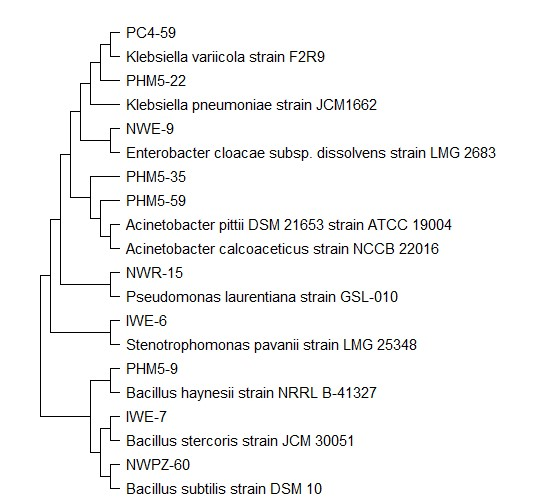

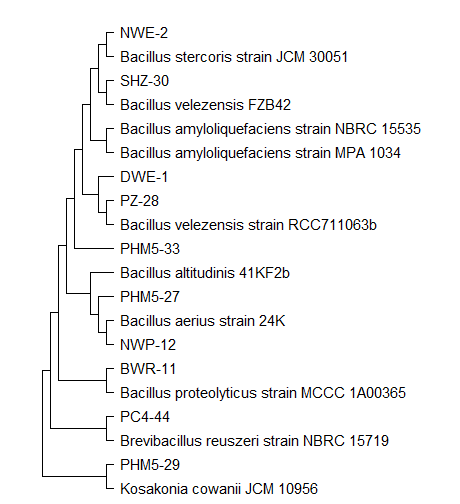

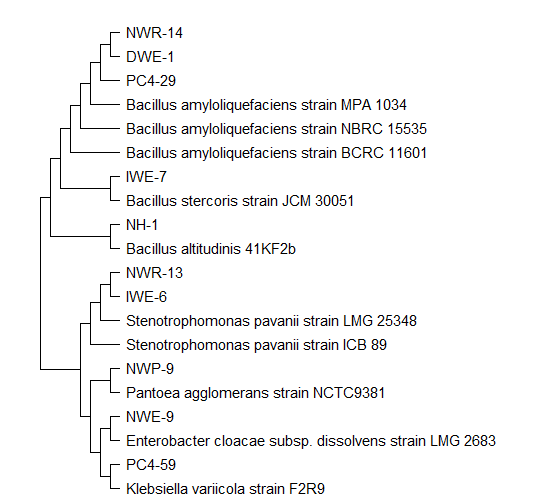


c

a

b

**Fig. S2. Phylogenetic tree based on 16S rRNA sequences of each community, a) for PGPts b) for CFts, and c) for PGPts+CFts**


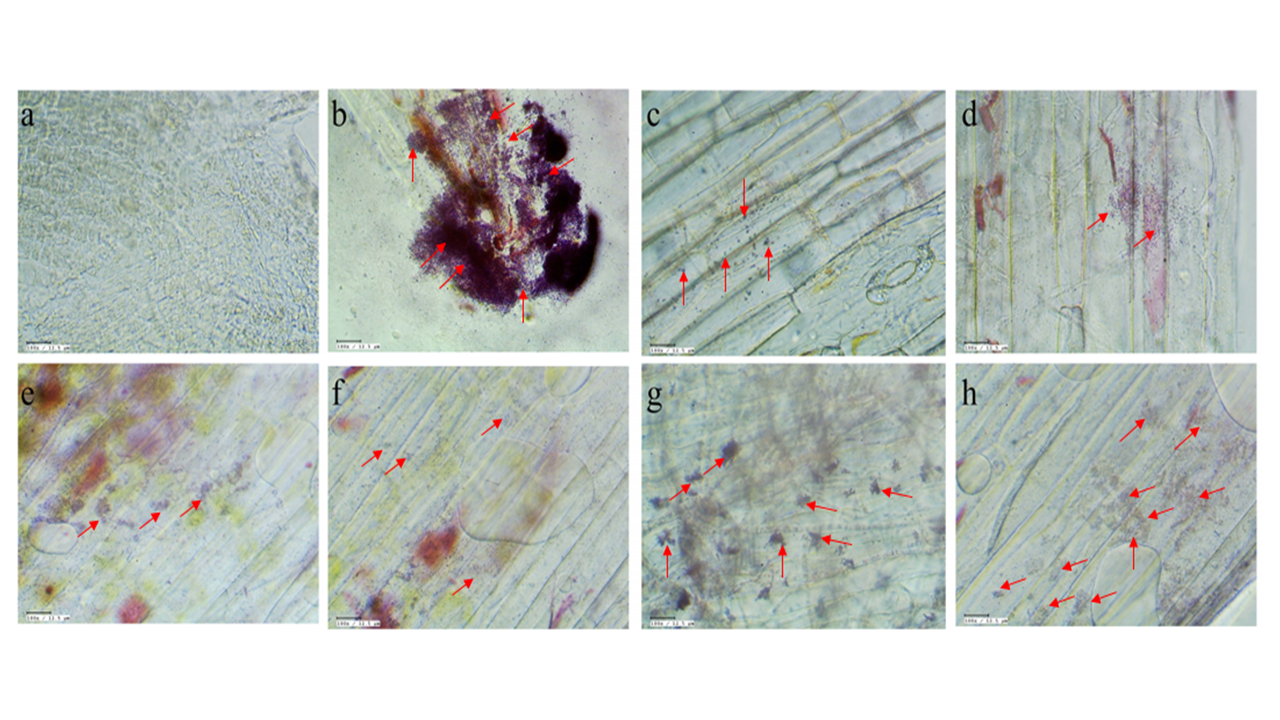


**Fig. S3. Colonization of bacterial community inside the wheat seedlings. a) Uninoculated seeds; Colonization in inoculated seeds after b)24h c)48h and d)72h; e) Colonization in leaf tissue; f), g), and h) Root colonization behavior of SM1, SM2 and SM3 bacterial communities**
