# Supplementary material for: Community-forming traits play role in effective colonization of plant-growth-promoting bacteria and improved plant growth
Source: Front Plant Sci. 2024 Mar 12;15:1332745. doi: 10.3389/fpls.2024.1332745 (PMC10963436; doi:10.3389/fpls.2024.1332745)
Supplement: Supplementary file 2 [file DataSheet_2.docx]

**Community Forming traits play role in effective colonization of Plant Growth Promoting bacteria and improved plant growth**

Devashish Pathak, Archna Suman*, Pushpendra Sharma, Aswini K, V Govindasamy, Shrikant Gond and Anshika

*Corresponding Author

Division of Microbiology, ICAR-Indian Agricultural Research Institute, New Delhi, India-110012

**Supplementary Table (S1). Selected isolates from different regions of the country with different morphotypes.**

| Region | Isolate | Shape | Colour | Margin | Texture | Slimy | Size |
| --- | --- | --- | --- | --- | --- | --- | --- |
| NASHIK | NWE1 | Irregular | Dull White | Regular | Smooth | No | Small |
|  | NWE2 | Irregular | White | Irregular | Smooth | Yes | Small |
|  | NWE3 | Circular | Cream | Regular | Smooth | Yes | Pinhead |
|  | NWE4 | Irregular | Cream | Irregular | Smooth | No | Medium |
|  | NWE5 | Irregular | White | Irregular | Smooth | No | Large |
|  | NWE6 | Irregular | White | Irregular | Smooth | Yes | Small |
|  | NWE7 | Circular | Dull White | Regular | Smooth | No | Large |
|  | NWE8 | Irregular | Cream | Irregular | Rough | No | Small |
|  | NWE9 | Irregular | Cream | Regular | Smooth | Yes | Large |
|  | NWE10 | Circular | White | Regular | Smooth | Yes | Large |
|  | NWE11 | Irregular | Dull White | Regular | Smooth | Yes | Small |
|  | NWR12 | Circular | Dull White | Regular | Smooth | No | Small |
|  | NWR13 | Irregular | Cream | Irregular | Rough | Yes | Medium |
|  | NWR14 | Irregular | Cream | Irregular | Rough | Yes | Pinhead |
|  | NWR15 | Circular | White | Regular | Smooth | Yes | Large |
|  | NWR16 | Circular | Dull White | Regular | Smooth | No | Small |
|  | NWR17 | Circular | White | Regular | Smooth | No | Medium |
|  | NWR18 | Irregular | Dull White | Irregular | Rough | No | Large |
|  | NWR19 | Irregular | Dull White | Irregular | Rough | No | Large |
|  | NWR20 | Circular | Dull White | Regular | Smooth | No | Small |
|  | NWR21 | Irregular | White | Irregular | Rough | No | Pinhead |
|  | NWR22 | Irregular | Cream | Irregular | Rough | No | Pinhead |
|  | NWR23 | Irregular | Dull White | Irregular | Rough | No | Pinhead |
|  | NWR24 | Circular | Dull White | Irregular | Smooth | No | Large |
| DELHI | DWE1 | Circular | Dull White | Regular | Smooth | Yes | Medium |
|  | DWE2 | Irregular | White | Irregular | Rough | No | Pinhead |
|  | DWE3 | Circular | Dull White | Regular | Smooth | Yes | Pinhead |
|  | DWE4 | Circular | Dull White | Regular | Rough | No | Large |
|  | DWE5 | Circular | Cream | Regular | Smooth | Yes | Medium |
|  | DWE6 | Irregular | White | Irregular | Rough | No | Medium |
|  | DWE7 | Irregular | White | Irregular | Smooth | Yes | Pinhead |
|  | DWE8 | Circular | Dull White | Regular | Smooth | No | Pinhead |
|  | DWR9 | Irregular | Dull White | Regular | Smooth | No | Small |
|  | DWR10 | Irregular | White | Regular | Smooth | No | Pinhead |
|  | DWR11 | Circular | Dull White | Regular | Smooth | No | Pinhead |
|  | DWR12 | Circular | White | Regular | Rough | Yes | Medium |
|  | DWR13 | Irregular | White | Irregular | Rough | Yes | Small |
|  | DWR14 | Irregular | Dull White | Irregular | Rough | No | Small |
| TAMIL NADU | TWE1 | Circular | Dull White | Regular | Smooth | No | Large |
|  | TWE2 | Irregular | Cream | Irregular | Rough | No | Medium |
|  | TWE3 | Irregular | White | Irregular | Rough | No | Small |
|  | TWE4 | Circular | Dull White | Regular | Smooth | No | Small |
|  | TWE5 | Irregular | Cream | Irregular | Rough | No | Large |
|  | TWE6 | Irregular | Cream | Irregular | Smooth | No | Large |
|  | TWR7 | Irregular | Cream | Irregular | Rough | No | Small |
|  | TWR8 | Irregular | Cream | Irregular | Rough | No | Medium |
|  | TWR9 | Circular | Dull White | Regular | Smooth | No | Medium |
|  | TWR10 | Irregular | White | Irregular | Smooth | Yes | Large |
|  | TWR11 | Irregular | Cream | Irregular | Rough | No | Medium |
|  | TWR12 | Circular | White | Regular | Smooth | No | Medium |
|  | TWR13 | Circular | Dull White | Irregular | Smooth | No | Pinhead |
|  | TWR14 | Circular | Dull White | Regular | Smooth | No | Small |
|  | TWR15 | Circular | Dull White | Regular | Smooth | No | Small |
|  | TWR16 | Irregular | White | Irregular | Smooth | No | Medium |
|  | TWR17 | Irregular | White | Irregular | Smooth | No | Small |
| INDORE | IWE1 | Irregular | White | Irregular | Rough | Yes | Pinhead |
|  | IWE2 | Irregular | Cream | Irregular | Rough | Yes | Small |
|  | IWE3 | Irregular | White | Irregular | Rough | No | Large |
|  | IWE4 | Circular | White | Regular | Smooth | No | Small |
|  | IWE5 | Irregular | Cream | Irregular | Rough | Yes | Pinhead |
|  | IWE6 | Circular | Cream | Regular | Rough | Yes | Pinhead |
|  | IWE7 | Irregular | Cream | Irregular | Rough | No | Medium |
|  | IWR8 | Circular | Cream | Regular | Smooth | No | Pinhead |
|  | IWR9 | Circular | Dull White | Regular | Smooth | Yes | Medium |
|  | IWR10 | Circular | White | Regular | Smooth | No | Large |
|  | IWR11 | Irregular | Cream | Irregular | Smooth | Yes | Small |
|  | IWR12 | Irregular | White | Irregular | Rough | No | Medium |
|  | IWR13 | Irregular | White | Irregular | Rough | No | Medium |
|  | IWR14 | Irregular | Dull White | Regular | Smooth | No | Pinhead |
|  | IWR15 | Irregular | Cream | Irregular | Rough | No | Medium |
|  | IWR16 | Circular | Dull White | Regular | Smooth | No | Large |
| SHIMLA | SWE1 | Irregular | Cream | Irregular | Rough | Yes | Large |
|  | SWE2 | Circular | White | Regular | Smooth | No | Medium |
|  | SWE3 | Irregular | White | Irregular | Smooth | No | Pinhead |
|  | SWE4 | Circular | White | Regular | Smooth | No | Small |
|  | SWE5 | Circular | Cream | Regular | Smooth | No | Pinhead |
|  | SWE6 | Irregular | White | Regular | Smooth | No | Medium |
|  | SWE7 | Circular | Dull White | Regular | Smooth | No | Large |
|  | SWE8 | Circular | White | Regular | Smooth | No | Pinhead |
|  | SWE9 | Circular | Dull White | Regular | Smooth | No | Small |
|  | SWE10 | Irregular | White | Irregular | Smooth | No | Medium |
|  | SWE11 | Circular | Dull White | Regular | Smooth | No | Large |
|  | SWE12 | Irregular | Cream | Irregular | Rough | No | Medium |
|  | SWE13 | Circular | White | Regular | Rough | Yes | Medium |
|  | SWE14 | Irregular | White | Irregular | Rough | No | Pinhead |
|  | SWR15 | Irregular | Cream | Irregular | Rough | No | Small |
|  | SWR16 | Circular | Dull White | Regular | Smooth | No | Large |
|  | SWR17 | Circular | Dull White | Regular | Smooth | No | Medium |
|  | SWR18 | Irregular | Dull White | Regular | Smooth | Yes | Pinhead |
| BIHAR | BWE1 | Circular | Cream | Regular | Smooth | Yes | Medium |
|  | BWE2 | Circular | Dull White | Regular | Smooth | No | Small |
|  | BWE3 | Circular | White | Regular | Smooth | No | Large |
|  | BWE4 | Irregular | Cream | Irregular | Rough | Yes | Small |
|  | BWE5 | Circular | Dull White | Regular | Smooth | No | Large |
|  | BWE6 | Circular | Dull White | Regular | Smooth | Yes | Pinhead |
|  | BWE7 | Irregular | White | Irregular | Rough | No | Medium |
|  | BWE8 | Irregular | White | Irregular | Rough | No | Large |
|  | BWR9 | Irregular | Cream | Regular | Smooth | No | Pinhead |
|  | BWR10 | Circular | Dull White | Regular | Smooth | No | Medium |
|  | BWR11 | Irregular | Cream | Irregular | Rough | Yes | Large |
|  | BWR12 | Circular | Dull White | Regular | Smooth | No | Pinhead |
|  | BWR13 | Irregular | White | Irregular | Smooth | No | Small |
|  | BWR14 | Circular | Dull White | Regular | Smooth | No | Large |
|  | BWR15 | Circular | White | Regular | Smooth | No | Pinhead |
|  | BWR16 | Circular | Dull White | Regular | Smooth | No | Pinhead |
|  | BWR17 | Irregular | Cream | Irregular | Rough | No | Medium |
|  | BWR18 | Circular | Dull White | Regular | Smooth | No | Medium |
